# Valorization of pulp and paper industry sludge to produce methane using different F/M ratios and its kinetics and toxicity analysis

**DOI:** 10.1038/s41598-026-48412-7

**Published:** 2026-04-20

**Authors:** Izharul Haq, Kiran V. Kottur, Ajay S. Kalamdhad

**Affiliations:** 1 Department of Biosciences, Manipal University Jaipur, Jaipur, 303007 Rajasthan India; 2 Department of Civil Engineering, Indian Institute of Technology Guwahati, Guwahati, 781039 India; 3 Integrated Materials Research Centre, Manipal University Jaipur, Jaipur, 303007 Rajasthan India

**Keywords:** Anaerobic digestion, Biochemical methane potential, Pulp and paper industry sludge, Food to microorganism ratio, Biogas production, Toxicity evaluation, Biotechnology, Environmental sciences

## Abstract

The pulp and paper industry (PPI) produces various lignocellulosic wastes, including paper sludge, which is rich in cellulose and suitable for biofuel production. Anaerobic digestion (AD) has emerged as a promising treatment technology due to its environmental and economic benefits. Biochemical methane production (BMP) assays were performed at different food-to-microorganisms (F/M) ratios: 1.0, 1.5, 2.0, 2.5, and a control (Inoculum). Among the four different F/M ratios, F/M 1.5 achieved the highest methane yield of 272 mL CH_4_/g VS, followed by 2.0 > 1.0 > 2.5 with methane yield of 250 > 247 > 227 mL CH_4_/g VS over a period of 42 days. The effect of the F/M ratio on specific methanogenic activity (SMA) was evaluated to support the interpretation of BMP assay results. Further effects of substrate biodegradability and methane production rate were also evaluated in a kinetic study using two simplified models, among them the modified Gompertz model provided the best fit (R^2^ = 0.997) to the experimental data. Statistical analysis using one-way ANOVA revealed that the F/M ratio significantly affected methane yield (p < 0.05). Fourier Transform Infrared Spectroscopy (FTIR) analysis confirmed the presence of active organic functional groups, which highlights the lignocellulosic degradation pattern before and after digestion. A phytotoxicity assay using *Vigna radiata* L. (mung bean) revealed a concentration-dependent decline in seed germination, shoot and root length, and biomass after digestion relative to the control. This study uniquely integrates F/M ratio optimization, biodegradability assessment, kinetic modeling, and statistical analysis to provide a complete understanding of methane production from PPI sludge. It also links process performance with environmental safety through phytotoxicity evaluation, offering a comprehensive waste-to-energy framework.

## Introduction

The rapid rise in population and technological development has significantly increased solid waste generation across numerous industries. It is ranked as the sixth most polluting industrial sector globally, generating large volumes of hazardous wastewater as a by-product of paper production^1,2^. The pulp and paper sector is highly water-intensive, consuming roughly 10–300 m^3^ of water per ton of product and generating more than 400 million tons of paper and cardboard globally each year. About three-quarters of the freshwater input leaves as heavily polluted wastewater, posing significant risks to both environmental quality and human health^3^. Globally, approximately 450 million tonnes of sludge are generated annually from PPIs^4^. This sludge contains inorganic, organic, and organometallic pollutants, including adsorbable organic halides (AOXs) at higher concentrations^5^.

Also, the wastewater generated may retain 40–45% of the original raw materials, including chlorophenols, insecticides, chlorinated phenols, chlorolignins, chlorinated resins, fatty acids, lignocellulosic compounds, and biocides^6^. In PPIs, primary sludge is produced after mechanical wastewater treatment and typically consists of fine fibres, fillers, metallic particles, sand, and coating materials. Secondary sludge (also referred to as activated sludge or bio-sludge) arises from microbial degradation processes used to remove organic matter, and its composition mainly includes lignin, proteins, hemicellulose, cellulose, and lipids. Primary and secondary sludges are often combined to form mixed sludge^7,8^, which poses significant environmental hazards. To reduce these impacts before discharge into water bodies, it typically undergoes several stages of treatment^9^. To promote sustainability, there is increasing demand for cost-effective, environmentally responsible sludge management solutions^10^.

Landfilling and incineration are the most commonly used methods for managing PPI sludge at present^11^. However, these strategies are becoming less sustainable due to rising operational expenses, increased leachate production, declining landfill availability, and growing concerns about groundwater pollution^12^. Consequently, PPI sludge is regarded as a highly promising feedstock for biofuel production, particularly bioethanol, due to its composition of short fibres, primarily cellulose and hemicelluloses, with up to 40%, along with substantial microbial biomass. Converting sludge into products like biofuels offers both environmental and economic benefits^13^. Among the various methods of volarization, AD stands out as a promising technology. It is one of the biological processes that degrade organic materials in the absence of oxygen to produce biogas, primarily composed of CH_4_ and CO_2_^14^. In addition to generating renewable energy, it also contributes to environmental sustainability by producing digestate, a nutrient-rich byproduct that can be applied as a biofertilizer in agricultural or forest ecosystems^15^.

Previous studies on AD of PPI sludge have mainly focused on methane potential or co-digestion strategies, with limited emphasis on process optimization and integrated assessment^16^. In contrast, the present study provides a structured evaluation of biodegradability and methane yield across varying F/M ratios using BMP assays, offering deeper insight into substrate utilization efficiency. The incorporation of kinetic modeling further strengthens the understanding of methane production dynamics. Additionally, this study uniquely links process performance with environmental safety by conducting phytotoxicity tests before and after digestion. This combined approach enables simultaneous assessment of energy recovery and digestate quality. Overall, its novelty lies in integrating F/M ratio optimization, biodegradability assessment, kinetic evaluation, statistical analysis, and digestate phytotoxicity into a single framework for sustainable waste-to-energy application.

## Materials and methods

### Substrate and inoculum

PPI sludge was collected regularly in 10 L high-density polyethylene containers from primary and secondary sludge tanks at Eco-Tech Papers, located in Kamalpur, Assam (26°21′12.14147" N, 91°40′29.57994" Ε). Fresh cow dung (FCD) was used as an inoculum, as it contains 30–70% obligate anaerobes among its microorganisms, along with abundant macro- and micronutrients that promote bacterial proliferation in anaerobic digestion^17^, sourced from a farm in Amingaon village near the IIT Guwahati campus. The collected PPI sludge and FCD were stored at 4 °C to preserve their physicochemical properties for subsequent analysis. The physicochemical characteristics of PPI sludge and FCD are shown in Table 1.

**Table 1:** Physico-chemical characteristics of PPI sludge and FCD.

S.No	Parameter	PPI sludge	FCD
1	pH	5 ± 0.03	7.2 ± 0.05
2	MC (%)	80.67 ± 1.93	78.66 ± 0.53
3	TS (%)	19.33 ± 1.93	21.33 ± 0.57
4	VS (%TS)	60.75 ± 1.70	76.5 ± 0.85
5	sCOD (mg/L)	22,272 ± 1015	26,545 ± 1100.0
6	VFA (mg/L)	550 ± 35	4760 ± 230.0
7	TOC (%)	33.39 ± 5.2	42.52 ± 32
8	TKN (%)	1.03 ± 0.01	0.53 ± 0.007
9	Lignin (%TS)	6.08 ± 0.11	–
10	Hemicellulose (%TS)	7.45 ± 0.25	–
11	Cellulose (TS%)	31.55 ± 0.55	–
12	C/N	32.41 ± 1.8	–
Heavy metals (mg/L extract)			
1	Fe	600.59	701.26
2	Zn	63.35	59.52
3	Cd	0.22	0.12
4	Mn	7.20	9.01
5	Cr	3.01	1.52
6	Ni	2.52	13.03
7	Pb	7.50	11.21
8	Cu	10.92	2.91

### Experimental setup of BMP assay

**Fig. 1:**
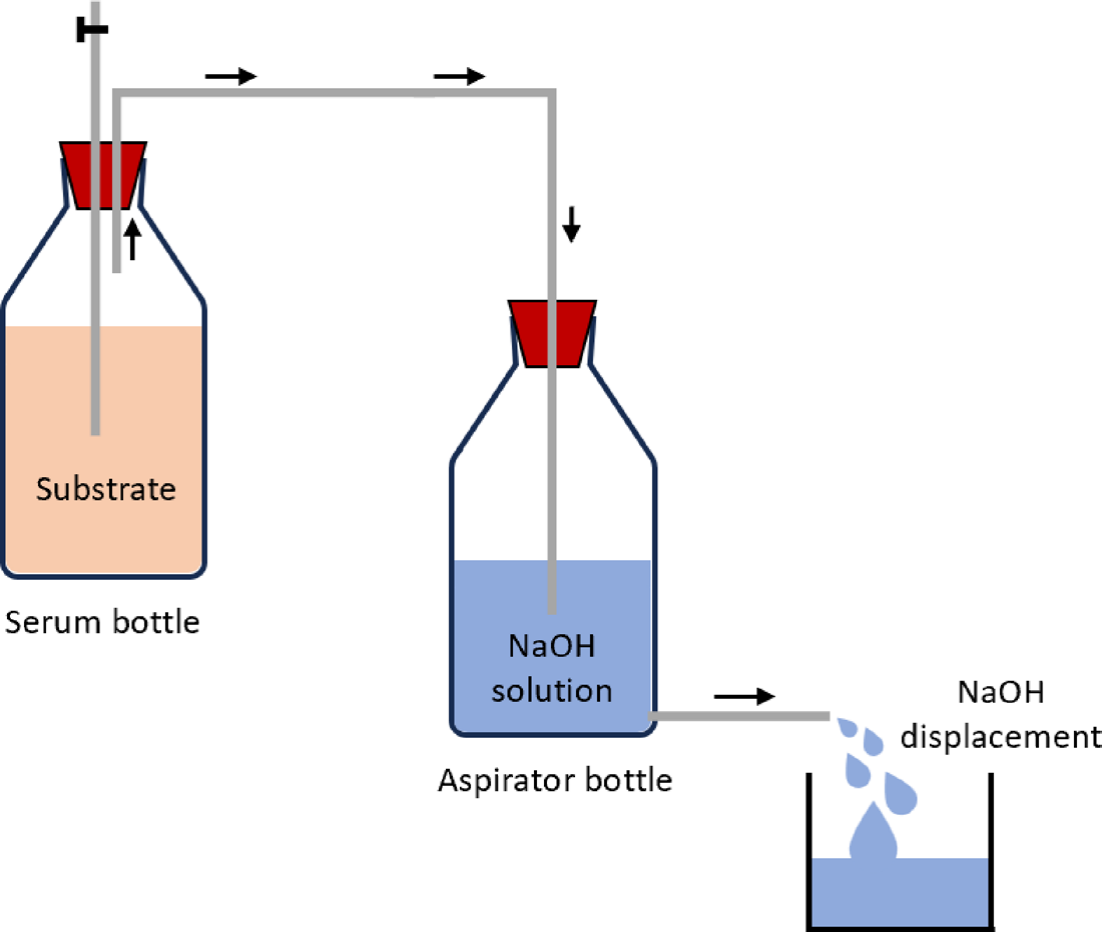
Schematic representation of BMP assay setup.

BMP assay assists in determining the maximum methane yield based on the volatile solids (VS) and soluble chemical oxygen demand (sCOD) content of organic matter^18^. In this study, BMP assay was conducted in a 1 L batch reactor with a working volume of 700 mL, operated under different F/M ratios (1.0, 1.5, 2.0, 2.5) and a control, at mesophilic conditions (35 ± 2 °C) over 42 days as illustrated in Fig. 1. A previous study by Li et al.^19^ evaluated the rate and extent of AD of cardboard waste at varying F/M ratios from 0.5 to 2.0. To promote optimal microbial activity and maintain adequate buffering capacity, reactors were supplemented with essential macro and micronutrients^20^. Methane production was quantified using the NaOH displacement method. Biogas was passed through an alkaline solution (1.5N NaOH) to selectively absorb CO_2_ and H_2_S, allowing direct measurement of methane volume. Gas volumes were recorded after pressure equalization and normalized to dry gas at standard temperature and pressure (0 °C, 1 atm). Methane yields were calculated after subtracting methane production from inoculum blanks and expressed as mL CH_4_/g VS removed. All tests were conducted in triplicate, and mixing was performed twice a day manually to ensure homogeneity. BMP assay was terminated when daily methane production was less than 1% of the cumulative methane yield for three consecutive measurements, in accordance with VDI 4630 guidelines.

The amounts of PPI sludge and FCD to be mixed were calculated on a VS basis using the following equation and are presented in Table 2.

$${\mathrm{F}}/{\text{M }} = \frac{{\text{(X)PPIS x (VS)PPIS}}}{{\text{(X)FCD x (VS)FCD}}}$$

where, (X)_PPIS_ = Amount of PPI sludge (g), (X)_FCD_ = Amount of Fresh cow dung (g) , (VS)_PPIS_ = Volatile solids in PPI sludge (%), (VS)_FCD_ = Volatile solids in Fresh cow dung (%).

**Table 2:** Feedstock composition for different F/M ratios.

F/M ratio	PPIS(g)	FCD(g)
1.0	125	100
1.5	187.5	100
2.0	250	100
2.5	312.5	100
Control	–	100

### Analytical study

The characterization of substrates was conducted to determine physicochemical parameters, including moisture content (MC), total solids (TS), and VS, following standard methods (APHA, 2017). pH of the samples was measured using a calibrated portable pH meter. Volatile fatty acids (VFAs) were quantified by pH titration and the short distillation method (DiLallo and Albertson, 1961). sCOD was assessed using the closed reflux titrimetric method (Standard methods 5220; APHA, 2017). Total Kjeldahl Nitrogen (TKN) was estimated by the Kjeldahl method, involving digestion, distillation, and titration steps. Total organic carbon (TOC) was determined using a TOC analyzer (Analytik Jena-Model, multi-N/C 2100s, Germany). Heavy metal concentration was analyzed using liquid chromatography coupled with inductively coupled plasma mass spectrometry (LC-ICP-MS; Agilent Model 7850 ICP-MS). Throughout the anaerobic biodegradability assay, samples were collected weekly from each reactor.

### Analysis of functional groups

FTIR was employed to evaluate and compare organic functional groups present in PPI sludge before and after pretreatment. For analysis, dried pretreated biomass at 1mg was thoroughly mixed with spectroscopic-grade potassium bromide (KBr) at 200mg using a mortar and pestle. The mixture was then pressed into transparent pellets at 10 MPa for 3 min. FTIR spectra were noted in the range of 4000 to 400cm^−1^, and an average of 16 scans per sample was used to ensure clean and representative data^21^.

### Kinetic study

To determine the extent and rate of biodegradation, kinetic experiments were performed. This study aims to evaluate the methane generation potential of different substrates using two commonly applied mathematical models: the First-order model and the modified Gompertz model. These models are useful for describing biodegradation kinetics and for estimating methane production from individual substrates or their mixtures^22^.

#### First-order model

The first-order model is a simplified tool that assumes methane production follows first-order kinetics^23^. According to this model, methane collection increases exponentially over time until it reaches a maximum. The mathematical expression of this model is given in Eq. (1).

1 $${\text{Y }} = {\text{ M }}\left[ {{1 } - {\text{ exp}}\left( { - {\mathrm{kt}}} \right)} \right]$$

where Y denotes cumulative methane produced (mL) at digestion time ‘t’ (days), M represents methane production potential (mL), and k is the growth rate constant (d^-1^).

#### Modified Gompertz model

The modified Gompertz model is obtained from the concept of natural exponential growth. It was originally proposed to trace the bacterial growth curves^24^ and has been widely applied by researchers to predict the performance of anaerobic digesters^25^. The mathematical form of this model is presented in Eq. 2.

2 $${\mathrm{Y}}_{{\left( {\mathrm{t}} \right)}} = {\mathrm{M}}{\text{ x exp}}\left\{ { - \exp \frac{{{(}{\text{Rm x e}}{)}}}{{\mathrm{M}}}(\lambda - {\text{ t}}) + 1} \right\}$$

where Y is cumulative methane produced (mL), M represents methane production potential (mL), R_m_ is maximum methane production rate (mL/day), λ denotes lag phase duration (days), and e is Euler’s number (2.7182).

### Statistical analysis

Analysis of Variance (ANOVA) is a widely used statistical tool to evaluate whether significant differences exist among the means of multiple groups under varying experimental conditions. In the present study, one-way ANOVA was used to assess the effects of operational parameters on methane yield and process performance using OriginPro 2025. A significance level of p < 0.05 is typically used to confirm statistically meaningful differences. All the experimental parameters were examined using descriptive statistics to verify the mean and standard deviations.

### Phytotoxicity assessment

The phytotoxicity of PPI sludge, before and after AD, was assessed using the method described by Haq and Kalamdhad^26^. For untreated sludge, 100g of sample was mixed with 300mL of ultrapure water and mechanically stirred for 20 h. The same homogenization procedure was applied to the digested sludge to maintain consistency across treatments. After stirring, the mixture was allowed to settle, and the resulting supernatant was collected for testing. The mung bean seeds used in the experiments were procured from a verified supplier in Guwahati, India. Prior to testing, the seeds were surface-sterilized by soaking in 0.1% (w/v) mercuric chloride solution for 10 min. Using deionized water, the remaining chemicals were thoroughly rinsed. Only healthy seeds were chosen and placed in sterile Petri dishes for assessment of germination and inhibition of sprout length^27^, OCED guidelines, 2003). By diluting the sludge supernatant with deionized water at varying concentrations ranging from 25 to 100% (v/v), test solutions were prepared. Petri dishes kept as controls were moistened using tap water. To monitor the seed germination, petri plates were incubated for 48 h at 28 °C in the dark. Following five days of incubation under a photoperiod regime, biomass accumulation and seedling growth were then evaluated at 8 h of light and 16 h of darkness.

## Results and discussions

### Physico-chemical characterization of PPI sludge and FCD

The PPI sludge predominantly comprises microbial biomass, proteins, carbohydrates, lignin, cellulose, hemicellulose, lipids, and various organic, inorganic, and heavy metal constituents^28^. Because of its plant-derived hemicellulose and lignin content, the sludge exhibits favourable characteristics for AD. The relatively high VS/TS ratio indicates a significant proportion of biodegradable organic matter, making it suitable for enhanced methane production. Although the pH was slightly below neutral, it remained within an acceptable range for optimal AD performance. The combination of a favourable VS/TS ratio and a significant sCOD concentration further supports the potential of the substrate for efficient methane yield. The quality and acceptability of the substrates were confirmed by the observed characteristics, which are reported in previous studies^21^.

### BMP test setup

F/M ratio optimization was conducted from 1.0 to 2.5, as F/M is a crucial factor for maximizing sludge digestion in a batch system, along with other influencing factors. The inoculum was essential for creating a balanced microbial population during the initial startup phase of the anaerobic system, especially syntrophic bacteria and methanogens. Preserving population balance is necessary to support syntrophic metabolism, which is thermodynamically advantageous during the AD process.

Different functional microbial groups are involved in a multi-step biochemical process that turns complex organic materials into biogas. Numerous microbial consortia, including hydrolytic bacteria, fermentative bacteria, homoacetogens (acetogens that produce or consume hydrogen), and methanogens (acetoclastic and hydrogenotrophic), play sequential roles in the breakdown of the organic components in PPI sludge^29^. These groups ensure the effective conversion of soluble and lignocellulosic organics into biogas.

#### Daily and cumulative methane production

**Fig. 2:**
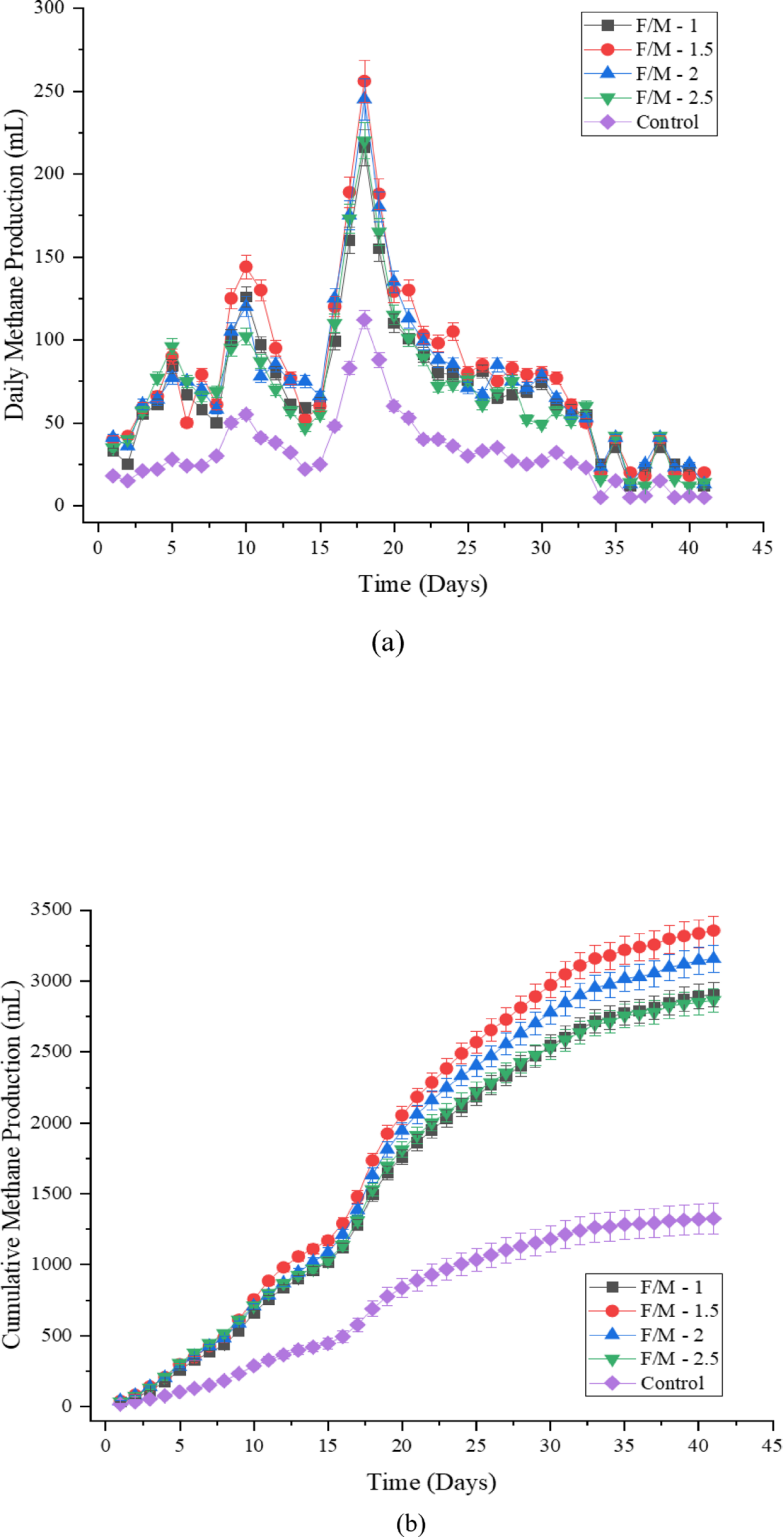
Variation in (**a**) Daily methane production and (**b**) Cumulative methane production at different F/M ratios on a daily basis.

The methane production rate serves as an indirect indicator of methanogenic biomass activity under anaerobic conditions. The observed trends in methane production were governed by sequential microbial pathways during the AD process. Initially, the hydrolytic bacteria degrade complex organic matter in sludge into soluble compounds, which are then converted into VFAs, hydrogen, and CO_2_ by acidogenic and acetogenic microbes. Methanogens subsequently utilize acetate and hydrogen to produce methane, making their activity crucial for process stability^30^. Any imbalance in these pathways, such as rapid acidogenesis leading to VFA accumulation, can inhibit methanogenesis and reduce methane yield. In this study, the PPI sludge demonstrated the highest methane yield of 272 mL CH_4_/g VS at an F/M ratio of 1.5, indicating this as the optimal loading condition. This was followed by an F/M ratio of 2.0 > 1.0 > 2.5 with methane yield of 250 > 247 > 227 mL CH_4_/g VS, respectively. A previous study by Priadi et al.^31^ reported a methane yield of 269 mL CH_4_/g VS, and a study by Veluchamy and Kalamdhad^21^ reported a methane yield of 264.5 mL CH_4_/g VS for pulp and paper industry sludge, which aligns with the methane yields obtained in the present study. Methane production trends were monitored both in terms of daily production rates and cumulative production, as illustrated in Fig. 2a and b, respectively. Given the lignocellulosic nature of PPI sludge, initial methane production was likely driven by the rapid degradation of the readily biodegradable volatile fraction. However, the significant solubilization phase commenced after 15 days and remained consistent through day 32 across all F/M ratios, consistent with reports indicating that hydrolysis of complex lignocellulosic components is the rate-limiting step during AD of such substrates.

#### VFAs

**Fig. 3:**
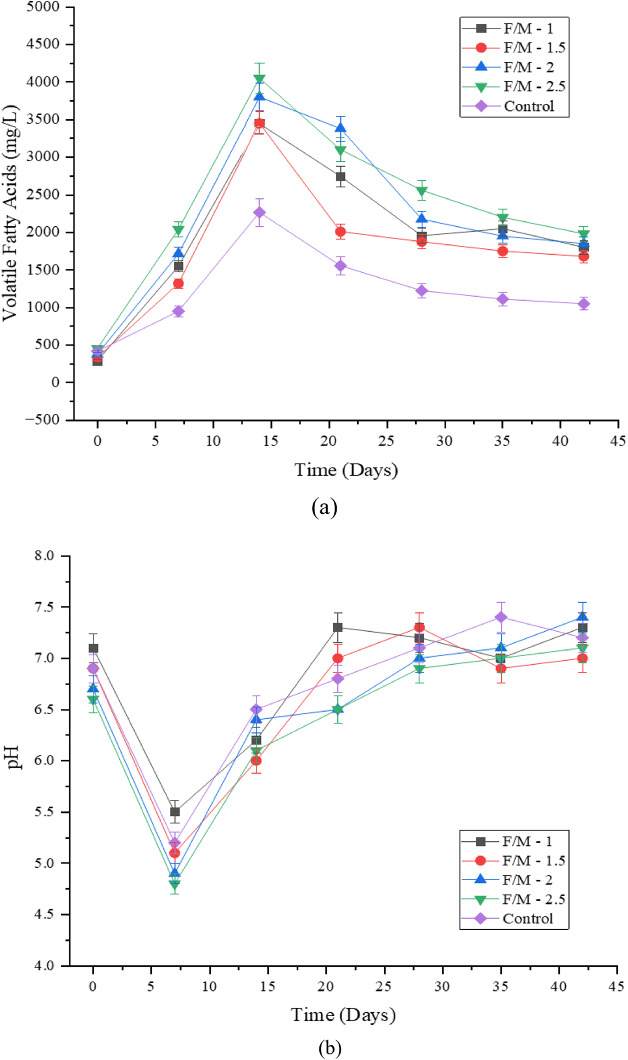
Variation in (**a**) VFA concentration and (**b**) pH at different F/M ratios on a weekly basis.

During methane production, F/M ratios ranging from 1.0 to 2.5 showed distinct patterns of VFA accumulation and degradation, as illustrated in Fig. 3a. Initially, hydrolytic bacteria activity released soluble organic compounds, which were subsequently fermented by acidogenic bacteria, resulting in increased VFA concentrations. These VFAs were later consumed by methanogens, leading to a gradual decline in VFA levels and an increase in biogas production^32^. The effect of F/M ratio on methane production is closely linked to microbial activity and system stability. At higher F/M ratios, excess substrate leads to rapid hydrolysis and acidogenesis, resulting in the accumulation of VFAs. This VFA buildup lowers pH and inhibits methanogenic microorganisms, reducing methane yield. In contrast, an optimal F/M ratio maintains a balance between acid production and consumption, ensuring efficient methane production^33^. During the early phase of digestion, all F/M ratios exhibit an increase in VFA concentration due to active acidogenesis. This accumulation caused a drop in pH, reflecting the metabolic dominance of acid-forming bacteria. However, after 14 days, a decline in VFA levels was observed, likely due to reduced substrate availability and the delayed induction of methanogenic enzymes. Excessive VFA accumulation led to a sharp pH reduction at an F/M ratio of 2.5, negatively affecting methanogenic activity and inhibiting methane production. A co-digestion study on lignocellulosic biomass by^34^ has reported similar results.

#### pH

An AD system includes a syntrophic relationship between acetogenic and methanogenic microorganisms, which is essential for maintaining process stability and optimal reactor performance. One of the simplest indicators of this microbial balance is pH, which reflects the dynamic interactions among various biochemical processes^35^. For efficient biogas production, it is critical to maintain the pH within the optimal range of 6.8 to 7.2. The pH in an AD system is influenced by the extent of organic matter degradation and solubilization, which are closely related to VFA and sCOD concentrations, bicarbonate alkalinity, and CO_2_ levels. In the present study, pH values within the reactor varied from 4.8 to 7.4, indicating that the PPI sludge substrate possessed a slightly acidic nature, as depicted in Fig. 3b. A sharp pH decline was observed during the initial phase of digestion primarily due to the rapid hydrolysis and acidogenesis of readily biodegradable organics, resulting in elevated VFA accumulation. However, as the process progressed, the pH gradually increased, indicating the start of methanogenesis and the subsequent consumption of VFAs. This transition marks the shift towards system stabilization.

#### sCOD

**Fig. 4:**
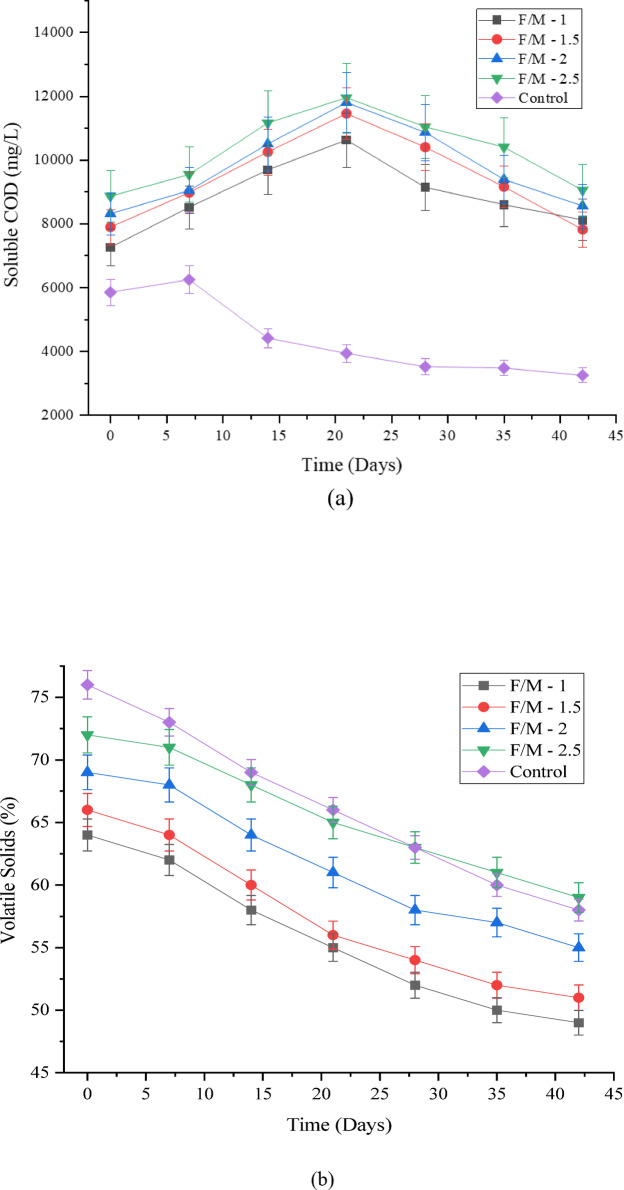
Variation in (**a**) sCOD concentration and (**b**) VS (%) at different F/M ratios on a weekly basis.

BMP assays performed at different F/M ratios showed distinct trends in sCOD levels. Figure 4a illustrates the sCOD profiles during an AD of PPI sludge under varying F/M conditions. During the initial stages of AD, acidogenic bacteria play a crucial role in solubilizing the organic fraction of PPI sludge. These microbes metabolize the soluble products generated during hydrolysis, such as sugars, amino acids, and fatty acids, into VFAs, alcohols, hydrogen, and CO_2_^36^. In addition, these microorganisms secrete extracellular enzymes that enhance the breakdown of complex lignocellulosic structures, increasing the availability of soluble substrates. This rapid conversion leads to an accumulation of VFAs in the early days of digestion, which reflects active acidogenesis and effective solubilization of organic matter^37^. As per Yu et al.^38^, an increase in sCOD indicates a greater quantity of readily transformable soluble organics. As digestion progressed, the accumulated organic acids were consumed by homoacetogens and methanogens, resulting in a decline in sCOD levels across all tested F/M ratios. sCOD concentrations generally peaked around day 14, then declined. This behaviour aligns with the finding reported by^39^. This study demonstrated a higher rate of solubility removal compared to other studies involving a similar type of industrial sludge^15^. The recalcitrant lignocellulosic compounds, including cellulose and lignin, underwent significant solubilization only after 21 days of digestion.

#### VS

VS reduction (%) is a key indicator of organic matter degradation and mass loss in an AD system, and it directly correlates with biogas production. In this study, the highest VS reduction was observed at an F/M ratio of 1.5, achieving 22.0%, corresponding to a maximum methane yield of 272 mL CH_4_/g VS. Compared with Veluchamy and Kalamdhad^21^, the present study demonstrates a significant reduction in VS%. The VS reduction (%) trend across the F/M ratio was as follows: 1.5 > 1.0 > 2.0 > 2.5, as shown in Fig. 4a. This trend indicates that moderate substrate loading facilitated optimal microbial performance and substrate degradation. A highly active inoculum led to relatively strong methane production, even though VS% reduction decreased significantly at higher F/M ratios. The VS reduction (%) reflects the conversion of volatile organic matter into methane and is quantified by conducting a mass balance for VS.

Preliminary VS mass balance.

The experimental result (VS removal) showed that 22.0% of soluble organic matter was converted during AD.

The theoretical relationship between VS removal and methane generation is:

$$1 g VS removed\approx 0.35{ L CH}_{4} (at STP)$$

Thus, VS removal directly supports and confirms that organic matter is converted into biogas.

Preliminary VS-based energy interpretation.

For F/M ratio – 1.5

Total solids = 8%

VS removed = 22%

Amount of sample = 700g.

VS removed = 0.08 × 0.22 × 700 = 12.32 g.

Theoretical methane yield: $12.32\times 0.35=4.31{ L CH}_{4} (\text{at STP})$

Based on the theoretical relationship of 0.35 L CH_4_/g VS removed, the observed VS removal was moderately consistent with the measured methane production.

### Effect of F/M ratio on SMA

**Fig. 5:**
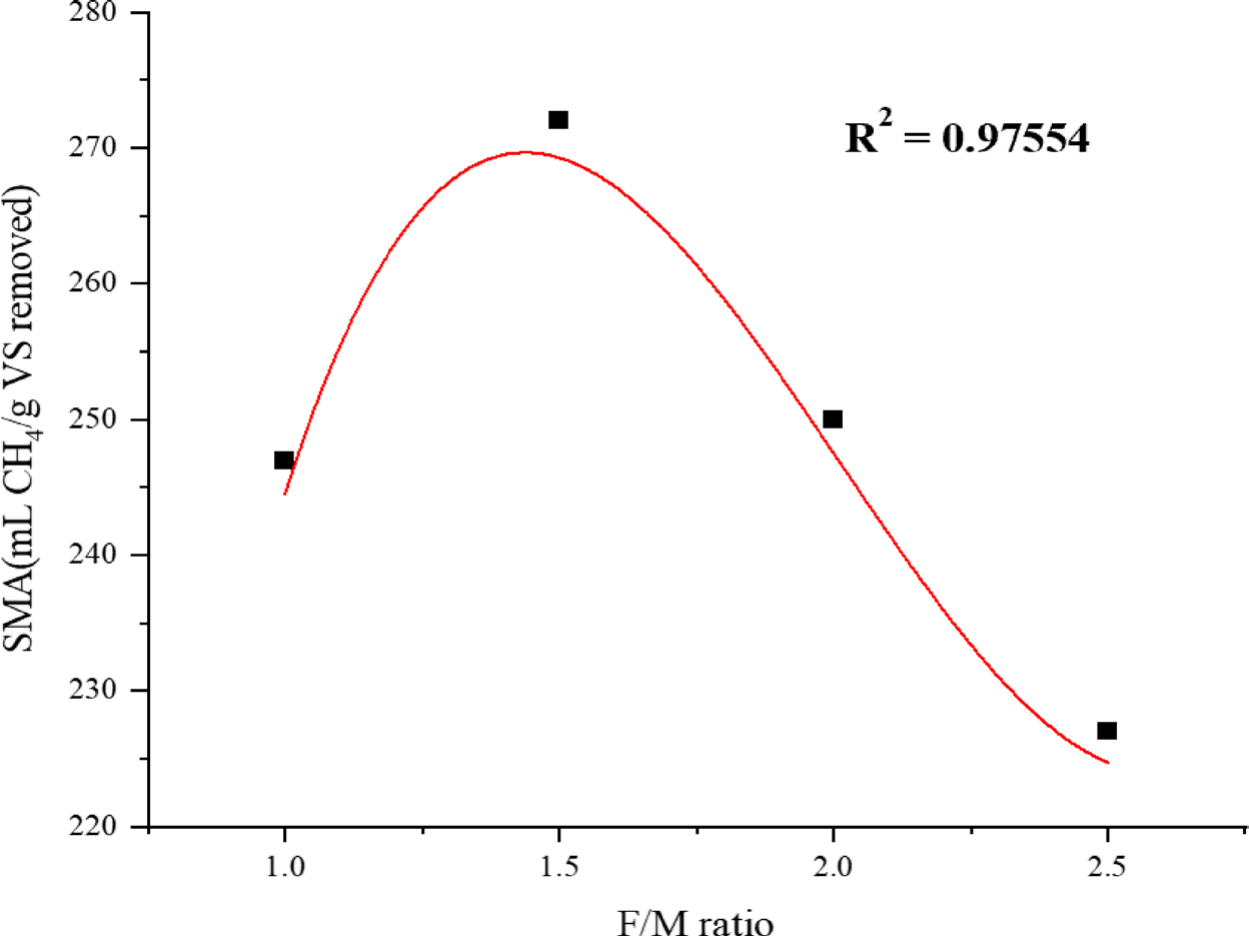
Effect of variation of F/M ratio on specific methanogenic activity of anaerobic digestion of PPI sludge.

The SMA after AD of PPI sludge initially increased from F/M 1.0 to 1.5 and then decreased. At lower (0.5) and higher (2.0 and 2.5) F/M ratios, SMA was lower, indicating that an optimal F/M ratio (1.5) enhances methanogenic activity and methane production (Fig. 5). Similar results have been reported by Hussain and Dubey^40^. The enhanced SMA at the optimal F/M ratio is attributed to the destabilization of lignocellulose and the increased availability of intracellular polymeric substances to methanogens.

### AD kinetics

**Fig. 6:**
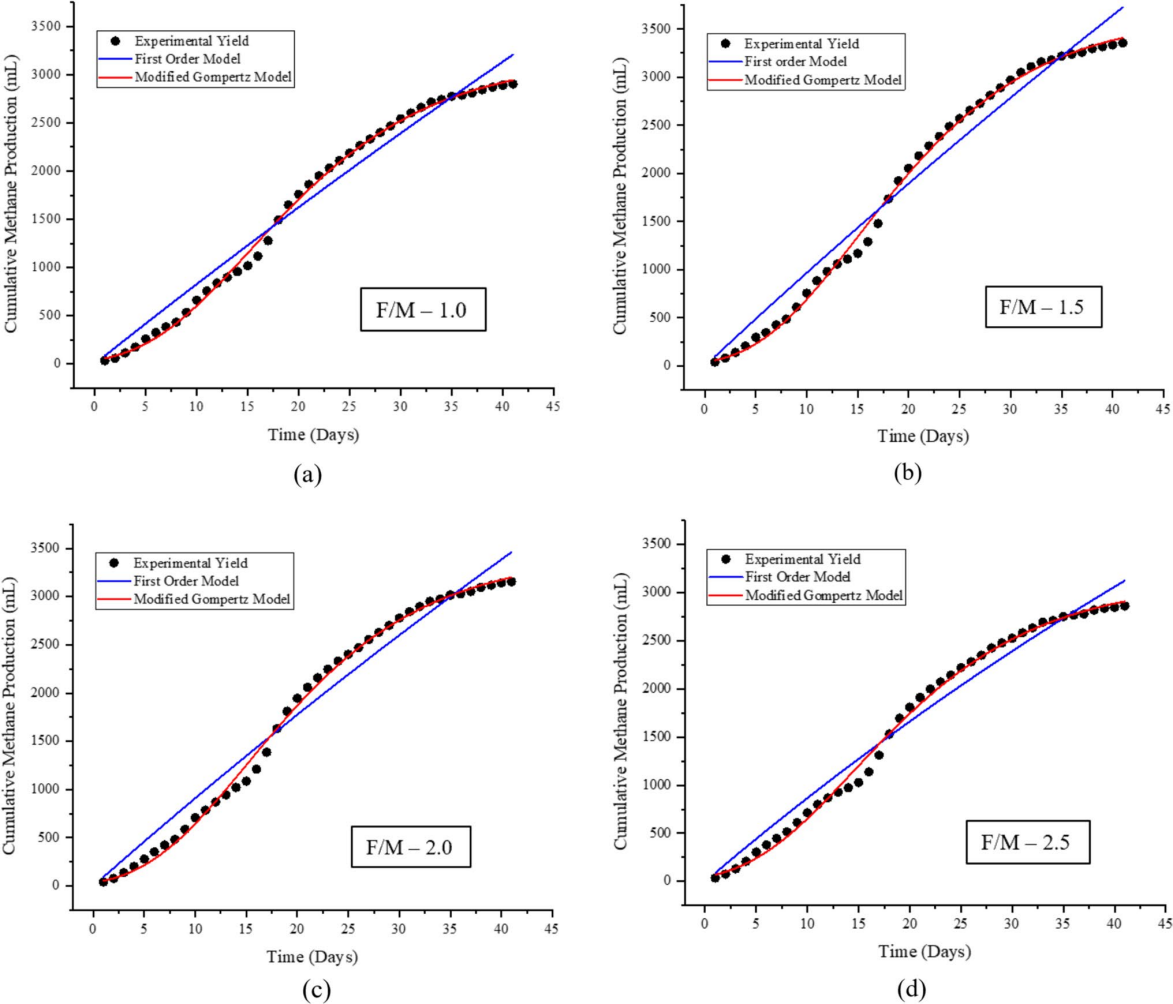
Kinetic analysis for cumulative methane production at F/M ratios of (**a**) 1.0, (**b**) 1.5, (**c**) 2.0, and (**d**) 2.5 using first-order model and modified Gompertz model.

Cumulative methane production data obtained from experiments were evaluated using the first-order and modified Gompertz models via a nonlinear regression approach. Kinetic analysis was performed using OriginPro 2025, and the parameters (Y_0_, λ, Rm, and k) were estimated for the respective models, as shown in Table 3. Both models provided reliable predictions of methane yield, with the first-order and modified Gompertz models showing percentage deviations of 6.41% and 9.27%, respectively, from the experimental value for the optimized F/M condition. The coefficient of determination (R^2^), which reflects the goodness of fit, for the first-order model and for the modified Gompertz model, is shown in Fig. 6a–d. The kinetic analysis shows that methane production increased with F/M ratio up to 1.5, where the highest methane potential (M = 3354 mL) and maximum production rate (R_m_ = 136 mL/day) were observed, indicating optimal microbial activity and substrate utilization. Beyond this point, at F/M ratios of 2.0 and 2.5, both M and R_m_ declined, suggesting substrate overloading and possible VFA accumulation leading to partial inhibition of methanogens. The lag phase (4.4–5.2 days) remained relatively consistent, indicating similar microbial adaptation time across conditions. The higher R^2^ values for the modified Gompertz model (0.995–0.997) compared to the first-order model confirm its better suitability in describing methane production kinetics^41^.

**Table 3:** AD Kinetics value of PPI sludge for different F/M conditions.

F/M ratio	M (mL)	R_m_ (mL/day)	λ (days)	Y (mL)	R^2^	Model
1.0	2904	–	–	3230	0.973	First Order
		115	5.015	3187	0.997	Modified Gompertz
1.5	3354	–	–	3682	0.969	First Order
		136	5.175	3663	0.997	Modified Gompertz
2.0	3155	–	–	3513	0.969	First Order
		127	5.185	3441	0.996	Modified Gompertz
2.5	2863	–	–	3262	0.973	First Order
		113	4.4	3135	0.995	Modified Gompertz

M = Experimental cumulative methane yield (mL).

Y = Predicted cumulative methane yield (mL).

R_m_ = Maximum methane production rate (mL/day).

λ = Lag phase (days).

R^2^ = Coefficient of determination.

The parameters (Y_0_, λ, Rm, and k) were discussed in terms of their relevance for preliminary assessment of substrate degradability and organic loading tolerance, while emphasizing that continuous or pilot-scale studies are required to translate these findings into reactor design and operational parameters.

### Analysis of variance

To evaluate the effect of different F/M ratios on methane yield, one-way ANOVA was performed. The results indicated that the F/M ratio had a statistically significant effect on methane production (p < 0.05). The high F-value suggests substantial variation between treatment groups compared to within-group variability. Post-hoc analysis using Tukey’s HSD test further confirmed that the methane yield at F/M ratio of 1.5 was significantly higher than other ratios, indicating an optimal F/M balance. The high R^2^ value (0.9917) indicates that variation in methane yield is strongly explained by changes in the F/M ratio. These findings confirm that the F/M ratio is a key operational parameter influencing AD performance. Table 4 presents the One-way ANOVA results for the effect of F/M ratio on methane yield during digestion.

**Table 4:** One-way ANOVA results showing the effect of F/M ratio on methane yield during AD process.

Parameter	Value	Interpretation
Test	One-way ANOVA	Comparison across F/M ratios
Factor	F/M ratio	Independent variable
Response variable	Methane yield	Dependent variable
F-value	719.74	Strong variation between groups
p-value	< 0.0001	Highly significant
R^2^	0.9917	Excellent model fit
Mean (Methane yield)	249 mL CH_4_/g VS	Average
SD (Methane yield)	18.42	Moderate variability

### FTIR analysis

**Fig. 7:**
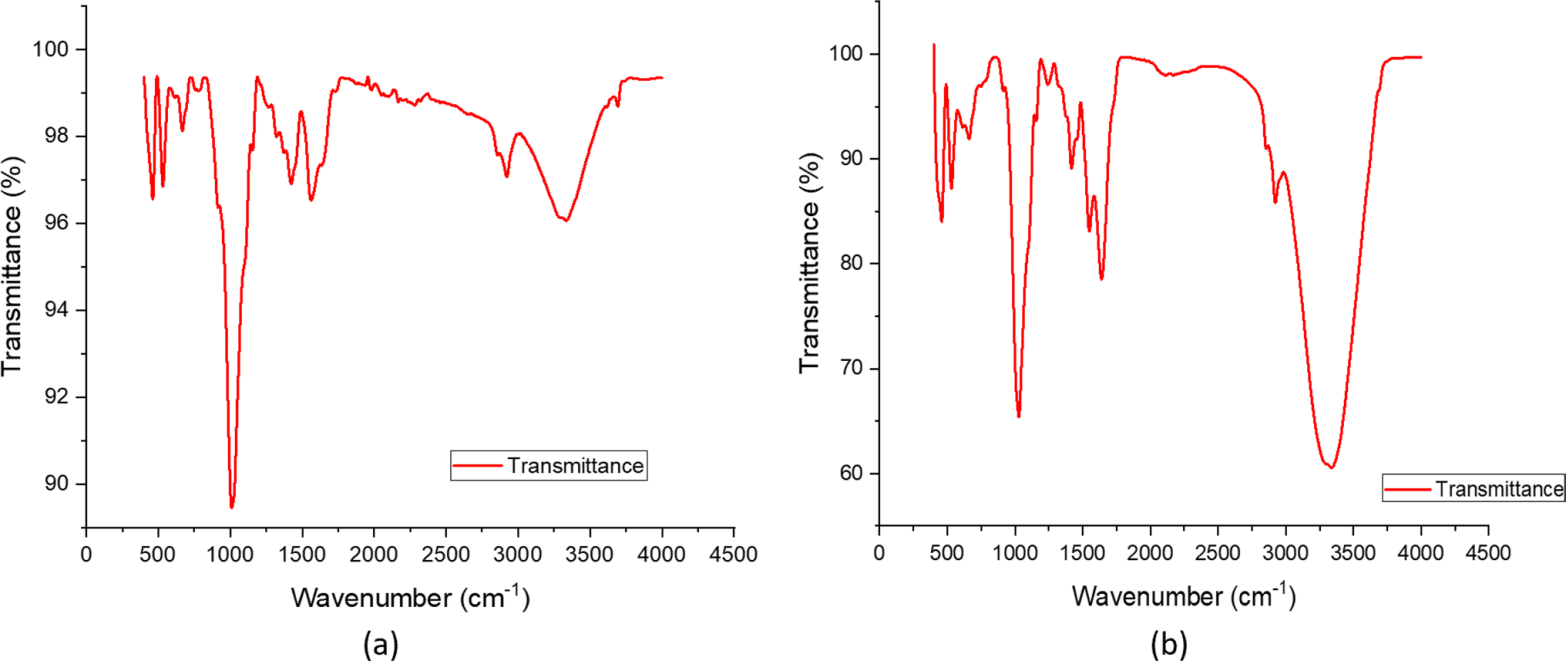
FTIR spectra variation in (**a**) Untreated and (**b**) Treated (digestate) chemical characteristics at optimal F/M condition.

In the present study, changes in the relative absorbance of these characteristic bands were utilized to assess the structural and lignocellulosic alterations in PPI sludge following AD, as depicted in Fig. 7. The FTIR spectra of untreated and treated sludge clearly demonstrate the degradation of lignocellulosic components during the AD process. In the untreated sludge, prominent peaks observed at 3200–3500 cm^−1^ (O–H stretching), 2900 cm^−1^ (C–H stretching), 1500–1700 cm^-1^ (aromatic C=C and C=0 of lignin), and 1000–1100 cm^−1^ (C–O stretching of cellulose and hemicellulose) confirm the presence of complex lignocellulosic structures^21^. After treatment, a significant reduction in peak intensity, particularly in the 1000–1100 cm^−1^ region, indicates the breakdown of polysaccharides, while a decreased intensity in the 1500–1700 cm^−1^ region suggests partial degradation of lignin. Changes in the 2800–3000 cm^−1^ region and the broadening of the 3200–3500 cm^−^1 peaks further reflect the transformation and solubilization of organic compounds. The FTIR results showing the spectral variations confirm effective hydrolysis and degradation of lignocellulosic biomass, enhancing the availability of simpler substrates for microbial conversion into methane.

### Phytotoxicity analysis

The comprehensive assessment of physical, chemical, and toxicity parameters provides a reliable basis for evaluating sludge quality prior to environmental discharge. Tap water was used as the control, representing a non-toxic, baseline aqueous medium with negligible organic load and trace levels of heavy metals. The physicochemical characteristics of untreated PPI sludge, digestate, and tap water were measured (Table 5), allowing direct comparison of pH, sCOD, and heavy metal levels across samples. Heavy metal concentrations in tap water were either below detection limits or present at trace levels well within drinking water guideline values, indicating that the control did not contribute to toxicity. The apparent increase in heavy metal concentration in digestate is mainly due to the reduction of total solids and organic matter during AD process, which concentrates the remaining non-degradable components. Since heavy metals are not degraded, their relative concentrations increase without additional input. This is a concentration effect rather than an actual rise in metal content or toxicity. PPI sludge samples were tested for phytotoxicity both before and after methane production. A well-known technique for assessing the toxicity of pesticides, sludge, or wastewater based on seed germination and sprout development is the phytotoxicity test, employing *Vigna radiata*L^26^..

**Table 5:** Physico-chemical characteristics of untreated PPI sludge, digestate, and tap water.

S.No	Parameter	Untreated	Digestate	Control (Tap water)
1	pH	6.9 ± 0.16	7 ± 0.1	7.15 ± 0.05
2	TS (%)	9.65 ± 0.75	3.28 ± 0.12	–
3	VS (%TS)	66.15 ± 1.5	44.2 ± 1.2	–
4	sCOD (mg/L)	7895 ± 350	5440 ± 280	–
5	VFA (mg/L)	335 ± 45	570 ± 60	–
6	Lignin (%TS)	6.08 ± 0.11	5.1 ± 0.15	–
7	Hemicellulose (%TS)	7.45 ± 0.25	2.45 ± 0.20	–
8	Cellulose (TS%)	31.55 ± 0.55	19.8 ± 0.35	–
Heavy metals (mg/L extract)				
1	Fe	150 ± 0.5	205 ± 1.22	0.055 ± 0.009
2	Zn	7.65 ± 0.05	8.05 ± 0.16	0.006 ± 0.001
3	Cd	0.075 ± 0.002	0.13 ± 0.011	ND
4	Mn	2.2 ± 0.025	3.5 ± 0.08	0.012 ± 0.002
5	Cr	1.05 ± 0.011	1.18 ± 0.01	ND
6	Ni	0.7 ± 0.001	0.9 ± 0.001	0.002 ± 0.001
7	Pb	2.5 ± 0.04	3 ± 0.055	0.030 ± 0.005
8	Cu	2.85 ± 0.03	2.9 ± 0.024	0.085 ± 0.011

ND = Not detected.

**Fig. 8:**
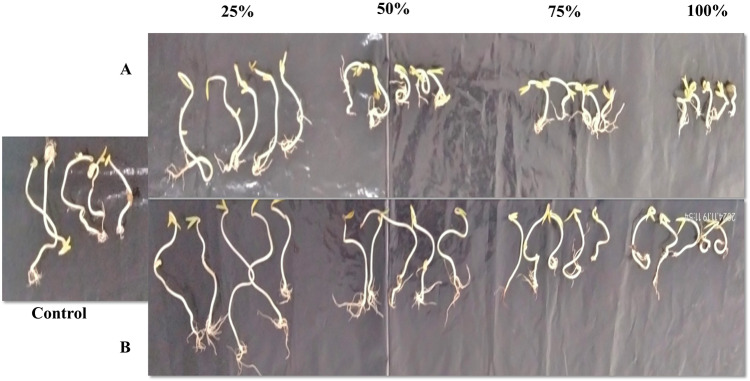
Effect of varying PPI sludge concentrations on mung bean (Vigna radiata L.) seedling growth (**A**) Before and (**B**) After anaerobic digestion.

In this study, the phytotoxic potential of PPI sludge at varying concentrations was assessed using mung bean seed germination tests over 48 h. Seed germination inhibition percentages across different concentrations are presented in Table 6. The control setup (tap water) exhibited no inhibition. However, at sludge extract concentrations up to 25%, germination inhibition was minimal in both untreated and treated samples. At 50% and 75% concentration of untreated sludge, seed germination inhibition reached 20% and 30%, respectively. The inhibition was reduced to 10% at 75% dilution of the treated sample. At 100% concentration of untreated PPI sludge, 40% inhibition of seed germination was recorded; post-digestion, the germination percentage increased to 80%. Figure 8 illustrates the impact of PPI sludge on seedling growth after 5 days. Maximum root growth in mung bean seedlings (2.74 ± 0.15 cm) was recorded at 25% concentration of untreated PPI sludge, while digested samples at the same concentration showed enhanced root elongation (4.261 ± 0.22 cm). At higher concentrations, a marked reduction in root length was observed in both treated and untreated PPI sludge samples, as shown in Table 6. A decrease in shoot length (2.96 ± 0.18 cm) was observed at 100% dilution of untreated PPI sludge, which was comparatively lower (6.32 ± 0.28 cm) in the digested sample at the same dilution when compared to the control. Similarly, mung bean seed biomass increased from 0.23 ± 0.04 mg in the untreated 100% PPI sludge sample to 0.37 ± 0.05 mg after treatment. The decrease in sCOD, TOC, and heavy metals resulting from the AD process may be linked to decreases in biomass, shoot length, root length, and seed germination (Sangeeta and Thangadurai, 2014^27^;) have examined crop responses to sludge, and these results are consistent with those findings. Osmotic stress and phytotoxicity may arise from decreased osmoregulation brought on by increased organic content, which may inhibit at higher digestive concentrations^42,43^. As observed in earlier studies on industrial sludge detoxification, decreases in biomass and root and shoot growth highlight the transformation of phenolics, hydrocarbons, and other organics during AD^27^. The observed reduction in phytotoxicity after AD was supported by concurrent decreases in sCOD and changes in heavy metal concentrations relative to the untreated sludge. These results suggest that toxicity reduction is primarily associated with organic matter degradation and stabilization during AD, with heavy metal levels remaining comparatively low in the control and not influencing the bioassay outcome^44^.

**Table 6:** Results of both untreated and treated PPI sludge samples on *V. radiata* L. seed germination, shoot length, root length, and seedling biomass growth.

Sludge	Dilution ratio (%)	Germination (%)	Shoot length (cm)	Root length (cm)	Biomass (gm)
Control		100 ± 4.1	10.96 ± 0.53	3.18 ± 0.12	0.91 ± 0.05
Untreated	25	90 ± 3.9	8.75 ± 0.41	2.74 ± 0.15	0.44 ± 0.03
	50	80 ± 4.2	5.87 ± 0.32	2.61 ± 0.13	0.40 ± 0.02
	75	70 ± 3.6	3.78 ± 0.17	2.26 ± 0.11	0.33 ± 0.02
	100	60 ± 3.2	2.96 ± 0.18	1.66 ± 0.07	0.73 ± 0.04
Digestate	25	100 ± 4.6	10.96 ± 0.57	4.26 ± 0.22	0.89 ± 0.03
	50	90 ± 4.1	9.44 ± 0.47	3.56 ± 0.17	0.73 ± 0.02
	75	90 ± 4.3	8.18 ± 0.39	3.28 ± 0.14	0.59 ± 0.06
	100	80 ± 3.9	6.32 ± 0.28	2.91 ± 0.16	0.37 ± 0.05

## Conclusion

There is significant potential to recover energy from PPI sludge as biogas. Four F/M ratios varying from 1.0 to 2.5 were examined on a VS basis. F/M ratio 1.5 showed the highest methane yield (272 mL CH_4_/g VS) among the studied F/M ratios. Kinetic analysis showed that the modified Gompertz model provided a better fit (R^2^ = 0.997) than the first-order model, focusing on the need for continuous studies to translate these findings into reactor design and operational parameters. At optimal conditions, the methane production rate (R_m_ = 136 mL/day) indicated optimal microbial activity, and the lag phase (λ = 5.175 days) suggested consistent microbial adaptation over time. The ANOVA results confirmed that the F/M ratio has a statistically significant influence on methane yield (p < 0.05), highlighting its importance in process optimization. The high F-value and R^2^ indicate that variations in methane production are strongly influenced by F/M ratio. The spectral variations observed in the FTIR results confirm effective hydrolysis and degradation of lignocellulosic biomass, thereby enhancing the availability of simpler substrates for microbial conversion into methane. The enhanced germination and seedling growth of mung beans indicate a significant reduction in phytotoxicity of the digestate following AD. Reduced phytotoxicity allows the digestate to be safely applied as an organic fertilizer in agricultural fields. It supports crop growth while promoting sustainable nutrient recycling and reducing reliance on chemical fertilizers. Thus, this study demonstrates a sustainable approach for the pulp and paper industry to transform highly polluted waste into a less toxic by-product while simultaneously generating renewable energy. Future efforts should focus on effective pretreatments, followed by continuous studies to enhance biodegradability and assess stability, loading rates, and retention times for translation to practical applications.

## Data Availability

All data, models or experimental frameworks used during the study are available from the corresponding author by request.
